# Bioactive Cembranoids from the Dongsha Atoll Soft Coral *Sarcophyton crassocaule*

**DOI:** 10.3390/md9060994

**Published:** 2011-06-09

**Authors:** Wan-Yu Lin, Yi Lu, Jui-Hsin Su, Zhi-Hong Wen, Chang-Feng Dai, Yao-Haur Kuo, Jyh-Horng Sheu

**Affiliations:** 1 Department of Marine Biotechnology and Resources, National Sun Yat-sen University, Kaohsiung 804, Taiwan; E-Mails: lemotylin@gmail.com (W.-Y.L.); snakefoot5052@gmail.com (Y.L.); wzh@mail.nsysu.edu.tw (Z.-H.W.); 2 National Museum of Marine Biology & Aquarium, Pingtung 944, Taiwan; E-Mail: x2219@nmmba.gov.tw; 3 Graduate Institute of Marine Biotechnology, National Dong Hwa University, Pingtung 944, Taiwan; 4 Institute of Oceanography, National Taiwan University, Taipei 112, Taiwan; E-Mail: corallab@ntu.edu.tw; 5 National Research Institute of Chinese Medicine, Taipei 112, Taiwan; E-Mail: kuoyh@nricm.edu.tw; 6 Division of Marine Biotechnology, Asia-Pacific Ocean Research Center, National Sun Yat-sen University, Kaohsiung 804, Taiwan

**Keywords:** soft coral, *Sarcophyton crassocaule*, cytotoxic activity, anti-inflammatory activity

## Abstract

Seven new cembranoids, sarcocrassocolides F–L (**1**–**7**), have been isolated from a soft coral *Sarcophyton crassocaule*. Their structures were determined by extensive spectroscopic analysis. Most new compounds exhibited significant cytotoxic activity against a limited panel of cancer cell lines, and the structure–activity relationship was studied. Compounds **1**–**7** were found to display significant *in vitro* anti-inflammatory activity in LPS-stimulated RAW264.7 macrophage cells by inhibiting the expression of the iNOS protein. Compound **4** was also found to effectively reduce the level of COX-2 protein.

## 1. Introduction

**Chart 1.**
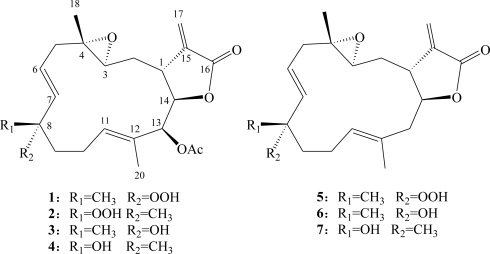
Structures of Metabolites **1**–**7**.

The cembrane-type compounds have been found to be the most important diterpenoidal constituents in marine coelenterates [1–8]. In the investigation of the bioactive metabolites from soft corals of Taiwanese waters, many bioactive cembranoids have been isolated from octocorals (Alcyonaceae) belonging to the genera *Sinularia* [9–15], *Lobophytum* [16,17], *Sarcophyton* [18–21] and *Pachyclavularia* [22,23]. Some of these metabolites have been shown to exhibit cytotoxic activity against the growth of various cancer cell lines [9,11,13,17–23], and/or anti-inflammatory activity [10–12,14–17]. Our recent study of the chemical constituents of the Dongsha Atoll soft coral *S. crassocaule* [24] has yielded cembranoids sarcocrassocolides A–E, which exhibited cytotoxic and anti-inflammatory activities. Our continuing chemical investigation of the dame collection of this organism, with the aim of discovering further biologically active natural products, again led to the isolation of seven new cembranoids, sarcrocrassocolides F–L (**1**–**7**) (Chart 1). The structures of **1**–**7** were established by extensive spectroscopic analysis, including careful examination of 2D NMR (^1^H-^1^H COSY, HMQC, HMBC and NOESY) correlations. The cytotoxicity of compounds **1**–**7** against human breast adenocarcinoma (MCF-7), human colon adenocarcinoma (WiDr), human laryngeal carcinoma (HEp-2) and human medulloblastoma (Daoy) cell lines was studied, and the ability of **1**–**7** to inhibit the up-regulation of pro-inflammatory iNOS (inducible nitric oxide synthase) and COX-2 (cyclooxygenase-2) proteins in LPS (lipopolysaccharide)-stimulated RAW264.7 macrophage cells was also examined.

## 2. Results and Discussion

**Figure 1:**
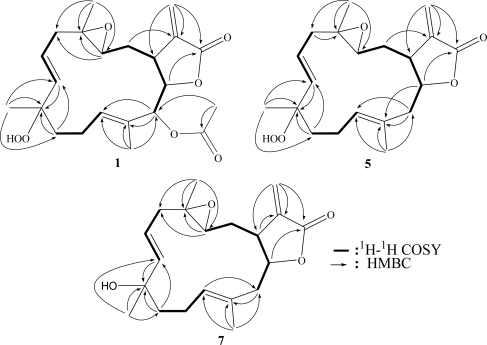
^1^H-^1^H COSY and HMBC correlations for **1**, **5** and **7**.

The HRESIMS (*m/z* 429.1887 [M + Na]^+^) of sarcrocrassocolide F (**1**) established the molecular formula C_22_H_30_O_7_, appropriate for eight degrees of unsaturation, and the IR spectrum revealed the presence of lactonic carbonyl (1757 cm^−1^) group. The ^13^C NMR and DEPT (Table 1) spectroscopic data showed signals of four methyls (including one acetate methyl), four sp^3^ methylenes, one sp^2^ methylenes, four sp^3^ methines (including three oxymethines), three sp^2^ methines, two sp^3^ and four sp^2^ quaternary carbons (including two ester carbonyls). The NMR signals (Tables 1 and 2) observed at δ_C_ 169.3 (qC), 139.3 (qC), 121.1 (CH_2_), 81.1 (CH), and 37.7 (CH), and δ_H_ 6.30, 5.64 (each, 1H, d, *J* = 2.5 Hz), 4.62 (1H, t, *J* = 3.0 Hz), and 3.10 (1H, dt, *J* = 12.0, 2.5 Hz) showed the presence of an α-methylene-γ-lactonic group by comparing the very similar NMR data of the cembranoids with the same five-membered lactone ring [18,19,24]. Signals resonating at δ_C_ 59.1 (qC), 59.2 (CH) and δ_H_ 2.57 (1H, dd, *J* = 6.5, 4.5 Hz) revealed the presence of a trisubstituted epoxide. The NMR signals at δ_C_ 84.4 (qC) and δ_H_ 7.42 (1H, brs) showed the presence of a hydroperoxy group at a methine carbon. One trisubstituted and one 1,2-disubstituted double bonds were also identified from NMR signals appearing at δ_C_ 128.7 (qC), 128.7 (CH), and δ_H_ 5.28 (1H, dd, *J* = 7.0, 1.0 Hz), and at δ_C_ 124.7 (CH), 136.4 (CH), and δ_H_ 5.49 (1H, dt, *J* = 16.0, 7.5 Hz) and 5.59 (1H, d, *J* = 16.0 Hz), respectively. In the ^1^H-^1^H COSY spectrum, it was possible to identify three different structural units, which were assembled with the assistance of an HMBC experiment. Key HMBC correlations of H_3_-18 to C-3, C-4 and C-5; H_3_-19 to C-7, C-8 and C-9; H_3_-20 to C-11, C-12 and C-13 and H_2_-17 to C-1, C-15 and C-16 permitted the establishment of the carbon skeleton (Figure 1). Furthermore, the acetoxy group positioned at C-13 was confirmed from the HMBC correlations of methyl protons of an acetate (δ_H_ 2.02) to the ester carbonyl carbon at δ_C_ 169.1 (qC) and the oxymethine carbon at 77.1 (C-13, CH). The *J* values for both H-6 and H-7 (16.0 Hz) further confirmed the presence of a *trans* 1,2-disubstituted double bond at C-6 and C-7. On the basis of the above analysis, the planar structure of **1** was established unambiguously.

**Figure 2:**
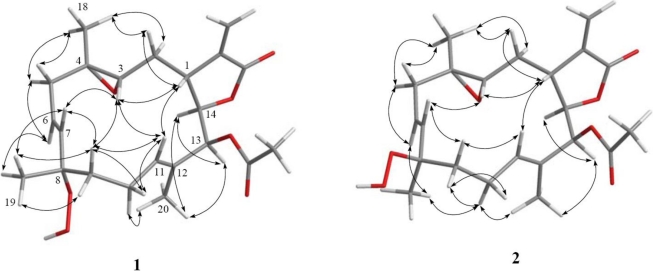
Key NOESY correlations for **1** and **2**.

The relative structure of **1** was elucidated by the analysis of NOE correlations, as shown in Figure 2. It was found that H-1 (δ 3.10, dt, *J* = 12.0, 2.5 Hz) showed NOE interactions with H-3 (δ 2.57, dd, *J* = 6.5, 4.5 Hz) and H-11 (δ 5.28, dd, *J* = 7.0, 1.0 Hz); therefore, assuming an β-orientation of H-1, H-3 should also be positioned on the β-face, and the epoxy oxygen should be placed on the α-face. NOE correlations of H-3 with H-11 and H-7 (δ 5.59, d, *J* = 16.0 Hz), but not with H_3_-18 (δ 1.30, s), reflected the *trans* stereochemistry of epoxide. The *E* geometry of the trisubstituted double bond at C-11 and C-12 was assigned from NOE correlations of H_3_-20 (δ 1.76, s) with H-10α (δ 2.39, ddt, *J* = 17.0, 10.5, 5.0 Hz), and H-11 with H-10β (δ 2.02, brs), in addition to the upper field chemical shift of C-20 (δ 15.2). H-14 (δ 4.62, t, *J* = 3.0 Hz) exhibited NOE correlations with both H-13 (δ 5.38, s) and H_3_-20, but not with H-1 and H-11, indicating the α-orientation of both H-13 and H-14. One of the methylene protons at C-9 (δ 1.37, dt, *J* = 10.0, 5.0 Hz) exhibited NOE correlations with all of H-3, H-10β, H-11, H_3_-19 (1.41, s) and H-7, thus this C-9 proton and H_3_-19 should be positioned on the β-face. On the basis of the above findings and detailed examination of other NOE correlations (Figure 2), the relative structure of compound **1** was determined.

Sarcrocrassocolide G (**2**) possessed the same molecular formula (C_22_H_30_O_7_) as that of **1**, as revealed from HRESIMS. Furthermore, it was found that the NMR spectroscopic data of **2** (Tables 1 and 2) were found to be close to those of **1**. The overlapping proton signals at δ_H_ 5.52 and 5.54, measured in CDCl_3_, was clearly resolved by measuring the ^1^H NMR spectrum in pyridine-*d*_5_ (see Experimental Section 3.3.2) into two mutually coupled proton (δ_H_ 5.63, dd, *J* = 16.0, 7.0 Hz and 5.89, d, *J* = 16.0 Hz), attributable to a *trans* 1,2-disubstituted double bond. The relative stereochemistry of **2** was determined by analysis of the NOESY spectrum of **1**, also measured in pyridine-*d*_5_ (Figure 2). From the NOESY spectrum, it was found that H_3_-19 (δ 1.55, s) showed NOE interaction with H-6 (δ 5.63, dt, *J* = 16.0, 7.0 Hz) and H-9a (δ 2.06, ddd, *J* = 10.0, 5.0, 3.5 Hz), but not with H-7, showing the β-orientation of H_3_-19. Further analysis of other NOE interactions revealed that **2** possessed the same relative configurations at C-1, C-3, C-4, C-13 and C-14, as those of **1** (Figure 2). Therefore, **2** was found to be the C-8 epimer of **1**.

Sarcrocrassocolide H (**3**) was shown by HRESIMS to possess the molecular formula C_22_H_30_O_6_ (*m*/*z* 413.1937 [M + Na]^+^). Comparison of the ^1^H and ^13^C NMR data (Tables 1 and 2) of compounds **1** and **3** showed that both compounds should have similar structures. C-8 of **3** showed signal at upperfield δ_C_ 72.9 relative to the corresponding signal of **1** (δ_C_ 84.4), implying the presence of a hydroxyl group at C-8 of **3**. Moreover, the reduction of **1** by triphenylphosphine afforded **3**. Thus, the structure of **3** was established. Sarcrocrassocolide I (**4**) was shown to possess the same planar structure as that of **3 by** ^1^H-^1^H COSY and HMBC correlations. In order to confirm the relative stereochemistry of **4**, a reduction of **2** was performed to afford **4**. Thus, **4** was found to be the C-8 epimer of **3**.

**Figure 3:**
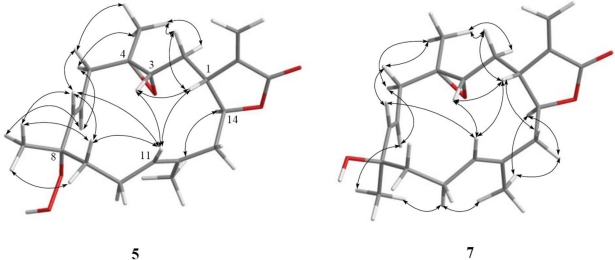
Key NOESY correlations for **5** and **7**.

Sarcrocrassocolide J (**5**) was shown by HRESIMS to possess the molecular formula C_20_H_28_O_5_ (*m*/*z* 371.1837 [M + Na]^+^). The IR spectrum of **5** showed the absorption of lactonic carbonyl (1760 cm^−1^) group. Comparison of the NMR data (Tables 1–3) of compounds **1** and **5** showed that the structure of **5** should be close to that of **1**, with the exception of signals assigned to C-13, where an acetoxymethine (δ_H_ 5.38, 1H, s; δ_C_ 77.1) in **1** was replaced by a methylene (δ_H_ 2.58, 1H, dd, *J* = 14.0, 5.0 Hz, δ_H_ 2.25, 1H, dd, *J* = 14.0, 8.0 Hz; δ_C_ 44.1) in **5**. The planar structure of **5** could be established by analyzing the ^1^H-^1^H COSY and HMBC correlations (Figure 1). The relative stereochemistry of **5** was confirmed by analyzing the key NOE correlations (Figure 3), and by comparison of these correlations with those of **1**. The structure of sarcocrassocolide J, as shown in formula **5**, was thus established.

Sarcrocrassocolide K (**6**) was shown by HRESIMS to possess the molecular formula C_20_H_28_O_4_ (*m*/*z* 355.1888 [M + Na]^+^). Comparison of the NMR data (Tables 1 and 3) of compounds **5** and **6** showed that both compounds have similar structures. Moreover, H_3_-19 (δ_H_ 1.32, s) and C-8 (δ_C_ 73.0) of **6** displayed signals at upper field in comparison with the corresponding signals of **5** (δ_H_ 1.40, H_3_-19; δ_C_ 84.9, C-8), showing the presence of a hydroxy group at C-8 of **6**. Furthermore, reduction of **5** with triphenylphosphine was found to give **6**. Thus, the structure of **6** was established.

Sarcrocrassocolide L (**7**) was shown by HRESIMS to possess the molecular formula C_20_H_28_O_4_ (*m*/*z* 355.1885 [M + Na]^+^). Comparison of the NMR data (Tables 1 and 3) of compounds **6** and **7** showed both compounds could be C-8 epimers. The planar structure of **7** was also confirmed by the ^1^H-^1^H COSY and HMBC correlations (Figure 1). The relative configuration of **7**, which should be the C-8 epimer of **6**, was determined by key NOE correlations (Figure 3).

**Figure 4:**
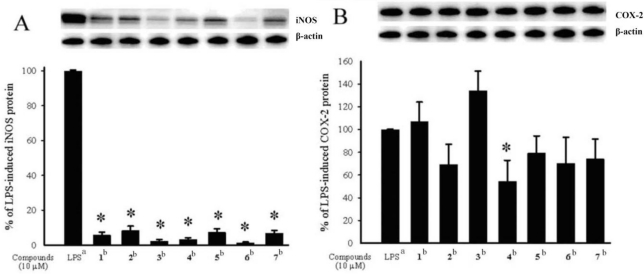
Effect of compounds **1**–**7** on iNOS and COX-2 proteins expression of RAW264.7 macrophage cells by immunoblot analysis: (**A**) Immunoblots of iNOS and β-actin; (**B**) Immunoblots of COX-2 and β-actin. The values are mean ± SEM (*n* = 6). Relative intensity of the LPS alone stimulated group was taken as 100 %.* Significantly different from LPS alone stimulated group (* *P* < 0.05). *^a^* stimulated with LPS, *^b^* stimulated with LPS in the presence of **1**–**7** (10 μM).

It is noteworthy to mention that metabolites **1**–**7** are cembranoids possessing an α-methylene-γ-lactonic group with a rarely found *trans* 6,7-disubstituted double bond, which has been discovered previously only in the soft coral *Eunicea pinta* [4]. These compounds could be the oxidized products of the related 7,8-olefinic analogues, although we have not yet successfully discovered that a cembranoid with the 7,8-double bond was converted into the corresponding 8-hydroxy or 8-hydroperoxy derivative under air in our laboratory. The cytotoxicity of compounds **1**–**7** against the proliferation of a limited panel of cancer cell lines, including Daoy, HEp-2, MCF-7 and WiDr carcinoma cell lines was evaluated. The results (Table 4) showed that compounds **1**–**4** were found to exhibit cytotoxicity against all or part of the above carcinoma cell lines, while compound **4** (ED_50_ values of 5.1 ± 1.2, 5.8 ± 0.5, 8.4 ± 1.5 and 6.4 ± 2.0 μM against above carcinoma cell lines, respectively) is the most potent one. Compound **5**, the 13-deacetoxy derivative of **1**, with ED_50_ values of >20 μM against above carcinoma cell lines and compound **7**, the 13-deacetoxy derivative of **4**, with ED_50_ values of >20 μM against above carcinoma cell lines, are less cytotoxic than **1** and **4**, respectively; therefore, it was suggested that the acetoxy group of C-13 is important for the cytotoxicity of compounds **1**–**7**. Compound **1** (ED_50_ value of 19.4 ± 2.4 μM against MCF-7 cells) which is the 8-peroxidized form of **3** (ED_50_ values of 9.4 ± 2.5 μM against MCF-7 cells), **2** (ED_50_ values of 8.3 ± 1.4, 16.5 ± 1.7 and 18.9 ± 1.9 μM against Daoy, HEp-2 and WiDr cells, respectively) which is the 8-peroxidized form of **4**, and **5** (ED_50_ values of >20 μM against Daoy, HEp-2 and WiDr cells) which is the 8-peroxidized form of **6**, are less cytotoxic than the corresponding 8-hydroxy derivatives **3**, **4** and **6**, respectively; therefore, it was found that the hydroxy group at C-8 could enhance the cytotoxicity of cembranoids **1**–**7**, in comparison with the C-8 hydroperoxy-bearing analogues. In the present study, the *in vitro* anti-inflammatory effects of compounds **1**–**7** were also tested by examining the inhibitory activity of these compounds toward the LPS-induced up-regulation of pro-inflammatory proteins, iNOS and COX-2 in RAW264.7 macrophage cells (Figure 4). At a concentration of 10 μM, compounds **1**–**7** were found to significantly reduce the levels of iNOS protein, relative to the control cells stimulated with LPS only. Furthermore, at the same concentration, metabolite **4** also could effectively reduce COX-2 expression with LPS treatment. Thus, compounds **1**–**7** might be useful anti-inflammatory agents, while **4** is a promising anti-inflammatory lead compound as **4** significantly inhibited the expression of both iNOS and COX-2 proteins. Compared to the biological activities of known cembranoids [9–24], **1**–**7** have shown satisfactory bioactivities and may warrant further study.

## 3. Experimental Section

### 3.1. General Experimental Procedures

Melting points were determined using a Fisher-Johns melting point apparatus. Optical rotations were measured on a JASCO P-1020 polarimeter. Ultraviolet spectra were recorded on a JASCO V-650 spectrophotometer. IR spectra were recorded on a JASCO FT/IR-4100 infrared spectrophotometer. The NMR spectra were recorded on a Varian 400MR FT-NMR (or Varian Unity INOVA500 FT-NMR) instrument at 400 MHz (or 500 MHz) for ^1^H and 100 MHz (or 125 MHz) for ^13^C in CDCl_3_. LRMS and HRMS were obtained by ESI on a Bruker APEX II mass spectrometer. Silica gel (Merck, 230–400 mesh) was used for column chromatography. Precoated silica gel plates (Merck, Kieselgel 60 F-254, 0.2 mm) were used for analytical TLC. High-performance liquid chromatography was performed on a Hitachi L-7100 HPLC apparatus with a Merck Hibar Si-60 column (250 × 21 mm, 7 μm) and on a Hitachi L-2455 HPLC apparatus with a Supelco C18 column (250 × 21.2 mm, 5 μm).

### 3.2. Animal Material

*S. crassocaule* (specimen no. 20070402), taxonomically identified by Prof. Chang-Feng Dai of National Taiwan University, was collected by hand using scuba off the coast of Dongsha, Taiwan, in April 2007, at a depth of 5–10 m, and stored in a freezer until extraction. A voucher sample was deposited at the Department of Marine Biotechnology and Resources, National Sun Yat-sen University.

### 3.3. Extraction and Separation

The frozen bodies of *S. crassocaule* (0.5 kg, wet wt) were minced and exhaustively extracted with EtOAc (1 L × 5). The EtOAc extract (7.3 g) was chromatographed over silica gel by column chromatography and eluted with EtOAc in *n*-hexane (0–100%, stepwise), then with acetone in EtOAc (50–100%, stepwise) to yield 28 fractions. Fraction 17, eluting with *n*-hexane–EtOAc (1:1), was further purified over silica gel using *n*-hexane–acetone (3:1) to afford seven subfractions (C1–C7). Subfraction C5 was purified by reverse-phase HPLC using MeOH–H_2_O (3:2) to afford **5** (2.2 mg). Subfraction C7 was separated by reverse-phase HPLC using MeOH–H_2_O (7:5) to afford **1** (6.3 mg) and **2** (9.3 mg). Fraction 18, eluting with *n*-hexane–EtOAc (1:1), and was further purified over silica gel using *n*-hexane–Acetone (3:1) to afford seven subfractions (D1–D7). Subfraction D3 was separated by reverse-phase HPLC using MeOH–H_2_O (3:2) to afford **6** (3.7 mg) and **7** (2.6 mg). Fraction 19, eluting with *n*-hexane–EtOAc (1:2), and was further purified over silica gel using *n*-hexane–Acetone (2:1) to afford seven subfractions (E1–E7). Subfraction E5 was separated by reverse-phase HPLC using MeOH–H_2_O (3:2) to afford **3** (5.2 mg) and **4** (4.3 mg).

Sarcocrassocolide F (**1**): white solid; mp 92.0–95.0 °C; [α]^25^_D_ −42 (*c* 0.6, CHCl_3_); IR (neat) ν_max_ 3403, 3014, 2974, 2933, 1757, 1659, 1430, 1371, 1273 and 1228 cm^−1^; UV (MeOH) λ_max_ 213 (log ɛ = 3.8); ^13^C and ^1^H NMR data, see Tables 1 and 2; ESIMS *m/z* 429 [M + Na]^+^; HRESIMS *m/z* 429.1887 [M + Na]^+^ (calcd for C_22_H_30_O_7_Na, 429.1889).

Sarcocrassocolide G (**2**): colorless oil; [α]^25^_D_ −56 (*c* 0.6, CHCl_3_); IR (neat) ν_max_ 3420, 2969, 2931, 2859, 1758, 1714, 1659, 1431, 1372, 1273 and 1229 cm^−1^; UV (MeOH) λ_max_ 209 (log ɛ = 3.7); ^13^C and ^1^H NMR data, see Tables 1 and 2; ^1^H NMR (Pyridine-*d*_5_, 500 MHz) δ 3.38 (1H, dt, *J* = 11.5, 3.0 Hz, H-1), 1.94 (1H, m, H-2a), 1.82 (1H, m, H-2b), 2.81 (1H, t, *J* = 6.0 Hz, H-3), 2.51 (1H, dd, *J* = 14.5, 7.0 Hz, 5a), 2.33 (1H, dd, *J* = 14.5, 7.0 Hz, 5b), 5.63 (1H, dt, *J* = 16.0, 7.0 Hz, H-6), 5.89 (1H, d, *J* = 16.0 Hz, H-7), 2.06 (1H, ddd, *J* = 10.0, 5.0, 3.5 Hz, H-9a), 1.97 (1H, dt, *J* = 13.5, 5.0 Hz, H-9b), 2.22 (2H, m, H_2_-10), 5.66 (1H, brs), 5.81 (1H, s), 4.91 (1H, t, *J* = 3.0 Hz, H-14), 6.45 (1H, d, *J* = 2.5 Hz, H-17a), 5.76 (1H, d, *J* = 2.5 Hz, H-17b), 1.30 (3H, s, H_3_-18), 1.55 (1H, s, H_3_-19), 1.80 (1H, s, H_3_-20), 2.03 (3H, s, 13-OAc), 13.04 (1H, s, 8-OOH); ^13^C NMR (Pyridine-*d*_5_, 125 MHz) δ 38.7 (CH, C-1), 35.7 (CH_2_, C-2), 59.3 (CH, C-3), 59.8 (qC, C-4), 39.6 (CH_2_, C-5), 124.6 (CH, C-6), 139.2 (CH, C-7), 84.7 (qC, C-8), 37.9 (CH_2_, C-9), 22.4 (CH_2_, C-10), 130.0 (CH, C-11), 130.2 (qC, C-12), 78.1 (CH, C-13), 82.6 (CH, C-14), 140.7 (qC, C-15), 169.9 (qC, C-16), 122.4 (CH_2_, C-17), 18.7 (CH_3_, C-18), 22.4 (CH_3_, C-19), 15.5 (CH_3_, C-20), 21.0 (CH_3_, C-OAc), 170.2 (qC, C-OAc); ESIMS *m/z* 429 [M + Na]^+^; HRESIMS *m/z* 429.1886 [M + Na]^+^ (calcd for C_22_H_30_O_7_Na, 429.1889).

Sarcocrassocolide H (**3**): colorless oil; [α]^25^_D_ −17 (*c* 0.5, CHCl_3_); IR (neat) ν_max_ 3479, 2966, 2927, 2856, 1758, 1659, 1432, 1370, 1273 and 1228 cm^−1^; UV (MeOH) λ_max_ 214 (log ɛ = 3.8); ^13^C and ^1^H NMR data, see Tables 1 and 2; ESIMS *m/z* 413 [M + Na]^+^; HRESIMS *m/z* 413.1937 [M + Na]^+^ (calcd for C_22_H_30_O_6_Na, 413.1940).

Sarcocrassocolide I (**4**): colorless oil; [α]^25^_D_ −29 (*c* 0.4, CHCl_3_); IR (neat) ν_max_ 3479, 2964, 2926, 2855, 1758, 1658, 1433, 1371, 1273 and 1230 cm^−1^; UV (MeOH) λ_max_ 212 (log ɛ = 3.7); ^13^C and ^1^H NMR data, see Tables 1 and 2; ESIMS *m/z* 413 [M + Na]^+^; HRESIMS *m/z* 413.1938 [M + Na]^+^ (calcd for C_22_H_30_O_6_Na, 413.1940).

Sarcocrassocolide J (**5**): colorless oil; [α]^25^_D_ −142 (*c* 0.1, CHCl_3_); IR (neat) ν_max_ 3382, 2961, 2928, 2857, 1760, 1659, 1431, 1384, 1268 and 1231 cm^−1^; UV (MeOH) λ_max_ 213 (log ɛ = 3.8); ^13^C and ^1^H NMR data, see Tables 1 and 3; ESIMS *m/z* 371 [M + Na]^+^; HRESIMS *m/z* 371.1837 [M + Na]^+^ (calcd for C_20_H_28_O_5_Na, 371.1834).

Sarcocrassocolide K (**6**): colorless oil; [α]^25^_D_ −51 (*c* 0.3, CHCl_3_); IR (neat) ν_max_ 3471, 2965, 2925, 2856, 1759, 1659, 1384 and 1266 cm^−1^; UV (MeOH) λ_max_ 208 (log ɛ = 3.7); ^13^C and ^1^H NMR data, see Tables 1 and 3; ESIMS *m/z* 355 [M + Na]^+^; HRESIMS *m/z* 355.1888 [M + Na]^+^ (calcd for C_20_H_28_O_4_Na, 355.1885).

Sarcocrassocolide L (**7**): white solid; mp 85–87 °C; [α]^25^_D_ −140 (*c* 0.2, CHCl_3_); IR (neat) ν_max_ 3445, 2965, 2925, 2854, 1759, 1654, 1455, 1374 and 1267 cm^−1^; UV (MeOH) λ_max_ 208 (log ɛ = 3.6); ^13^C and ^1^H NMR data, see Tables 1 and 3; ESIMS *m/z* 355 [M + Na]^+^ ;HRESIMS *m/z* 355.1883 [M + Na]^+^ (calcd for C_20_H_28_O_4_Na, 355.1885).

Reduction of sarcocrassocolide F (**1**). A solution of **1** (1.0 mg) in diethyl ether (3 mL) was added excess amount triphenylphosphine and the mixture was stirred at room temperature for 4 h. The solution was concentrated under reduced pressure to afford a residue which was subjected to reversed-phase HPLC with MeOH–H_2_O (3:2) to yield **3** (0.8 mg, 83%).

Reduction of sarcocrassocolide G (**2**). By using the same reaction and purification procedures as the reduction of **1**, the solution of **2** (1.0 mg) was converted to **4** (0.7 mg) in 73% yield.

Reduction of sarcocrassocolide J (**5**). By using the same reaction and purification procedures as the reduction of **1**, the solution of **5** (0.5 mg) was converted to **6** (0.4 mg) in 84% yield.

### 3.4. Cytotoxicity Testing

Cell lines were purchased from the American Type Culture Collection (ATCC). Cytotoxicity assays of compounds **1**–**7** were performed using the MTT [3-(4,5-dimethylthiazol-2-yl)-2,5-diphenyltetrazolium bromide] colorimetric method [25,26].

### 3.5. *In Vitro* Anti-Inflammatory Assay

Macrophage (RAW264.7) cell line was purchased from ATCC. *In vitro* anti-inflammatory activities of compounds **1**–**7** were measured by examining the inhibition of lipopolysaccharide (LPS) induced upregulation of iNOS (inducible nitric oxide synthetase) and COX-2 (cyclooxygenase-2) proteins in macrophages cells using western blotting analysis [27,28].

## Figures and Tables

**Table 1:** ^13^C NMR data for compounds **1**–**7**.

	**1***^a^*	**2***^b^*	**3***^b^*	**4***^b^*	**5***^a^*	**6***^a^*	**7***^a^*
1	37.7, CH *^c^*	38.7, CH	38.2, CH	38.2, CH	41.7, CH	41.6, CH	41.4, CH
2	35.2, CH_2_	35.2, CH_2_	36.0, CH_2_	34.5, CH_2_	32.9, CH_2_	32.9, CH_2_	32.3, CH_2_
3	59.2, CH	58.7, CH	59.2, CH	59.1, CH	59.6, CH	59.1, CH	59.4, CH
4	59.1, qC	59.0, qC	59.4, qC	59.5, qC	60.2, qC	60.1, qC	60.5, qC
5	38.6, CH_2_	38.6, CH_2_	39.2, CH_2_	38.8, CH_2_	40.1, CH_2_	39.8, CH_2_	39.8, CH_2_
6	124.7, CH	125.0, CH	121.2, CH	121.5, CH	125.7, CH	121.8, CH	121.0, CH
7	136.4, CH	136.9, CH	138.9, CH	140.7, CH	136.1, CH	140.1, CH	140.9, CH
8	84.4, qC	85.2, qC	72.9, qC	73.1, qC	84.9, qC	73.0, qC	73.0, qC
9	38.7, CH_2_	37.1, CH_2_	42.3, CH_2_	42.0, CH_2_	37.7, CH_2_	44.6, CH_2_	41.6, CH_2_
10	22.0, CH_2_	21.4, CH_2_	22.0, CH_2_	21.8, CH_2_	21.7, CH_2_	22.1, CH_2_	22.2, CH_2_
11	128.7, CH	128.3, CH	128.1, CH	130.1, CH	130.6, CH	131.1, CH	130.5, CH
12	128.7, qC	129.3, qC	127.7, qC	128.6, qC	129.1, qC	129.1, qC	129.2, qC
13	77.1, CH	77.3, CH	76.4, CH	77.4, CH	44.1, CH_2_	44.6, CH_2_	43.3, CH_2_
14	81.1, CH	81.4, CH	81.3, CH	82.0, CH	81.3, CH	82.3, CH	82.8, CH
15	139.3, qC	139.1, qC	138.1, qC	139.0, qC	139.0, qC	139.0, qC	139.1, qC
16	169.3, qC	169.3, qC	167.4, qC	169.4, qC	169.6, qC	169.6, qC	169.7, qC
17	121.1, CH_2_	122.2, CH_2_	121.1, CH_2_	121.8, CH_2_	122.4, CH_2_	122.4, CH_2_	122.0, CH_2_
18	18.2, CH_3_	18.2, CH_3_	19.3, CH_3_	18.0, CH_3_	17.6, CH_3_	17.6, CH_3_	17.6, CH_3_
19	23.5, CH_3_	20.9, CH_3_	31.2, CH_3_	28.4, CH_3_	22.3, CH_3_	29.8, CH_3_	29.4, CH_3_
20	15.2, CH_3_	15.2, CH_3_	16.4, CH_3_	14.6, CH_3_	17.0, CH_3_	16.9, CH_3_	17.2, CH_3_
OAc	20.8, CH_3_	20.8, CH_3_	21.8, CH_3_	20.8, CH_3_			
	169.1, qC	169.3, qC	167.4, qC	169.3, qC			

a Spectra recorded at 125 MHz in CDCl_3_;

b Spectra recorded at 100 MHz in CDCl_3_;

c Deduced from DEPT.

**Table 2:** ^1^H NMR data for compounds **1**–**4**.

	**1***^a^*	**2***^b^*	**3***^b^*	**4***^b^*
1	3.10 dt (12.0, 2.5) *^c^*	3.13 dt (11.6, 2.4)	3.11 dt (11.6, 2.8)	3.04 ddd (11.2, 4.4, 2.4)
2	1.84 ddd (14.5, 4.5,2.5) *^c^*	1.86 m	1.82 ddd (15.2, 5.6, 2.8)	1.85 m
	1.69 ddd (14.5, 12.0, 7.0)	1.71 m	1.72 m	1.74 m
3	2.57 dd (6.5, 4.5)	2.58 dd (6.8, 4.8)	2.59 t (5.6)	2.64 t (6.4)
5	2.48 dd (14.5, 7.5)	2.50 dd (15.2, 6.4);	2.46 t (2.8)	2.51 dd (14.4, 6.4)
	2.27 dd (14.5, 7.5)	2.30 dd (15.2, 6.4)	2.24 t (2.4)	2.21 dd (11.6, 14.4)
6	5.49 dt (16.0, 7.5)	5.52 dt (16.0, 6.4)	5.51 m	5.51 ddd (16.0, 8.0, 6.4)
7	5.59 d (16.0)	5.54 d (16.0)	5.49 m	5.60 d (16.0)
9	2.22 ddd (14.5, 10.5, 5.0)	1.91 m	2.04 brs	1.87 m
	1.37 dt (10.5, 5.0)	1.56 m	1.45 m	1.60 m
10	2.39 ddt (17.0, 10.5, 5.0)	2.04 m	2.34 m	2.15 m
	2.02 brs		2.05 brs	2.07 m
11	5.28 dd (7.0, 1.0)	5.30 brs	5.30 d (8.4)	5.41 m
13	5.38 s	5.37 s	5.38 s	5.40 brs
14	4.62 t (3.0)	4.57 t (2.8)	4.60 t (2.8)	4.59 dd (4.4, 2.4)
17	6.30 d (2.5)	6.32 d (2.4)	6.31 d (2.0)	6.30 d (2.4)
	5.64 d (2.5)	5.67 d (2.4)	5.65 d (2.0)	5.62 d (2.4)
18	1.30 s	1.31 s	1.30 s	1.32 s
19	1.41 s	1.35 s	1.34 s	1.30 s
20	1.76 s	1.73 s	1.75 s	1.71 s
8-OOH	7.42 s			
13-OAc	2.02 s	2.03 s	2.02 s	2.04 s

a Spectra recorded at 500 MHz in CDCl_3_;

b Spectra recorded at 400 MHz in CDCl_3_;

c *J* values (Hz) in parentheses.

**Table 3:** ^1^H NMR data for compounds **5**–**7**.

	**5***^a^*	**6***^a^*	**7***^a^*
1	2.80 ddd (10.5, 5.0, 3.0) *^c^*	2.79 ddd (10.5, 5.5, 3.0)	2.84 ddd (10.5, 5.5, 2.5)
2	1.83 ddd (15.5, 10.5, 5.5)	1.90 m	1.83 m
	1.78 ddd (15.5, 7.0, 3.0)	1.75 ddd (14.5, 7.5, 3.0)	
3	2.66 dd (7.0, 5.5)	2.71 dd (7.5, 4.5)	2.71 t (6.0)
5	2.60 dd(14.0, 5.0)	2.58 m	2.58 dd (15.0, 6.0)
	2.15 dd (14.0, 6.5)	2.15 ddd (26.0, 10.5, 3.0)	2.12 dd (15.0, 8.0)
6	5.57 ddd (16.0, 6.5, 5.0)	5.53 m	5.53 ddd (16.0, 8.0, 6.0)
7	5.58 d (16.0)	5.55 d (16.0)	5.61 d (16.0)
9	2.04 m	1.91 m	1.73 m
	1.51 m	1.57 m	
10	2.34 dt (13.5, 8.0)	2.28 d (8.0)	2.19 m
	2.02 m	2.06 m	2.09 m
11	5.23 brs	5.24 t (6.5)	5.29 t (7.0)
13	2.58 dd (14.0, 5.0)	2.59 dd (15.0, 5.5)	2.55 dd (15.0, 6.0)
	2.25 dd (14.0, 8.0)	2.26 d (8.0)	2.35 dd (15.0, 6.0)
14	4.49 dt (8.0, 5.0)	4.47 dt (8.0, 5.5)	4.47 q (6.0)
17	6.32 d (2.5)	6.33 d (2.5)	6.31 d (3.0)
	5.62 d (2.5)	5.63 d (2.5)	5.60 brs
18	1.32 s	1.33 s	1.34 s
19	1.40 s	1.32 s	1.30 s
20	1.67 s	1.67 s	1.65 s
13-OAc			

a Spectra recorded at 500 MHz in CDCl_3_;

b Spectra recorded at 400 MHz in CDCl_3_;

c *J* values (Hz) in parentheses.

**Table 4:** Cytotoxicity of compounds **1**–**7** (ED_50_ μM).

**Compound**	**Daoy**	**HEp-2**	**MCF-7**	**WiDr**
1	7.3 ± 1.7	15.0 ± 1.9	19.4 ± 2.4	18.4 ± 0.9
2	8.3 ± 1.4	16.5 ± 1.7	9.6 ± 2.7	18.9 ± 1.9
3	6.4 ± 2.0	13.5 ± 2.5	9.4 ± 2.5	18.7 ± 1.0
4	5.1 ± 1.2	5.8 ± 0.5	8.4 ± 1.5	6.4 ± 2.0
5	>20	>20	>20	>20
6	9.9 ± 4.0	>20	10.2 ± 1.0	>20
7	>20	>20	>20	>20
Mitomycin-C	0.44 ± 0.06	0.30 ± 0.06	0.30 ± 0.12	0.47 ± 0.12
