# Supplementary material for: Bioactive Cembranoids from the Dongsha Atoll Soft Coral Sarcophyton crassocaule
Source: Mar Drugs. 2011 Jun 9;9(6):994–1006. doi: 10.3390/md9060994 (PMC3131557; doi:10.3390/md9060994)
Supplement: Supplementary file 1 [file marinedrugs-09-00994-s001.pdf]

Supporting Information

Bioactive Cembrane-Based Diterpenoids from the Soft Coral *Sinularia triangular*

Jui-Hsin Su* and Zhi-Hong Wen

Table of Contents

S1. Table of Contents

S2. ^{1}H NMR spectrum of **1** in $CDCl_3$ at 400 MHz.

S3. ^{13}C NMR spectrum of **1** in $CDCl_3$ at 100 MHz.

S4. HMQC spectrum of **1** in $CDCl_3$.

S5. HMBC-1 spectrum of **1** in $CDCl_3$.

S6. HMBC-2 spectrum of **1** in $CDCl_3$.

S7. ^{1}H–^{1}H COSY spectrum of **1** in $CDCl_3$.

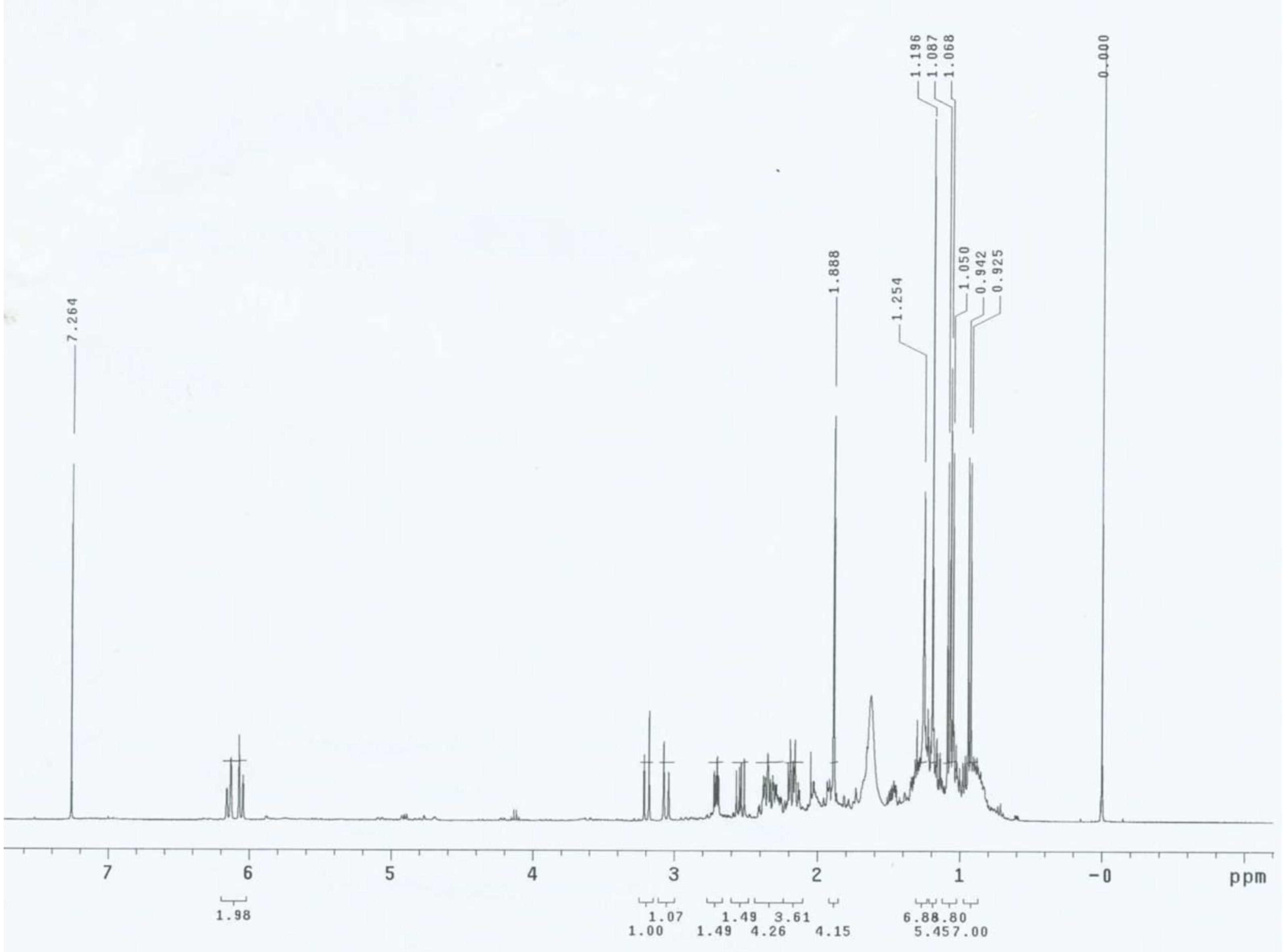

S2. ^{1}H NMR spectrum of **1** in $CDCl_3$ at 400 MHz.

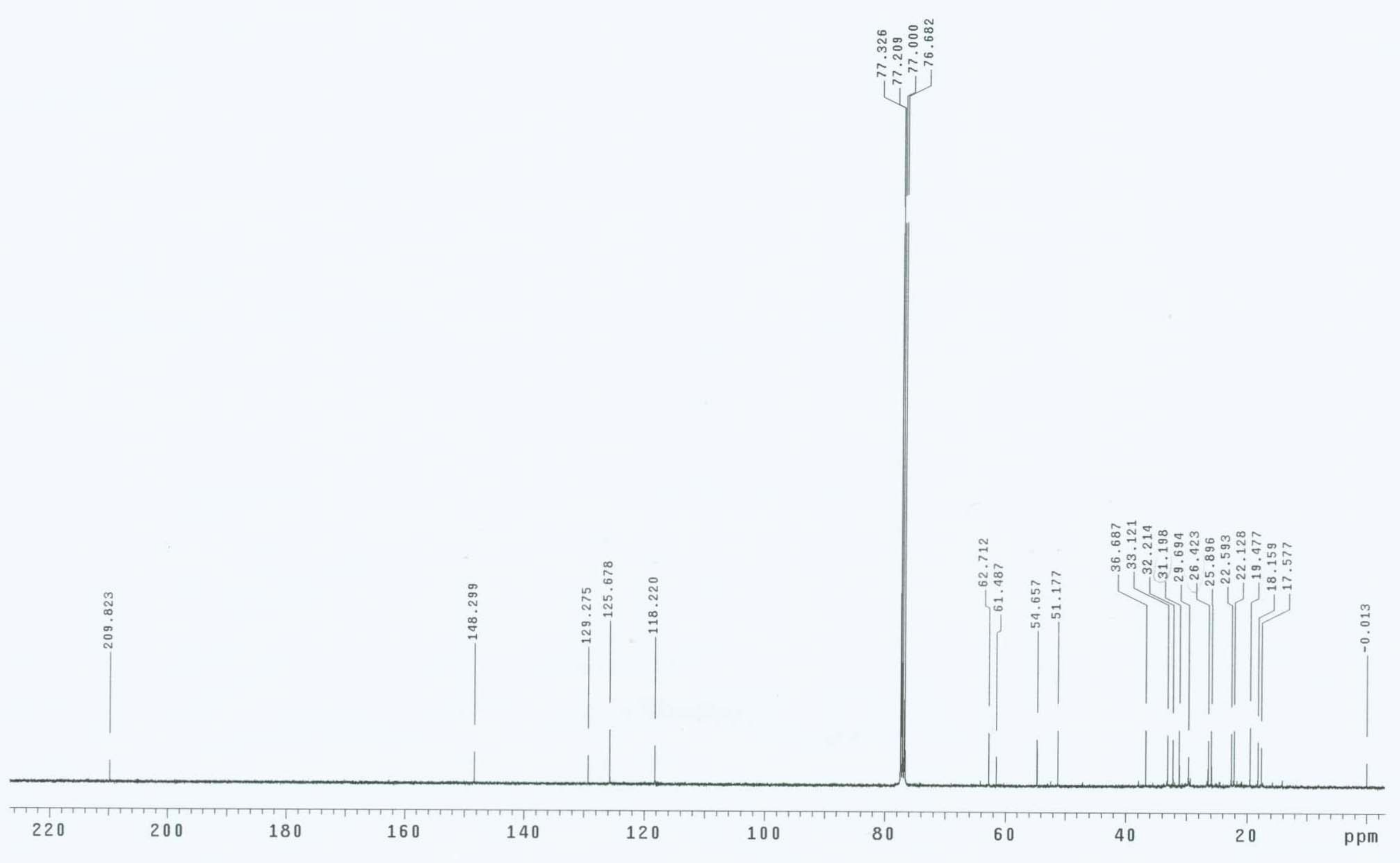

S3. ^{13}C NMR spectrum of **1** in $CDCl_3$ at 100 MHz.

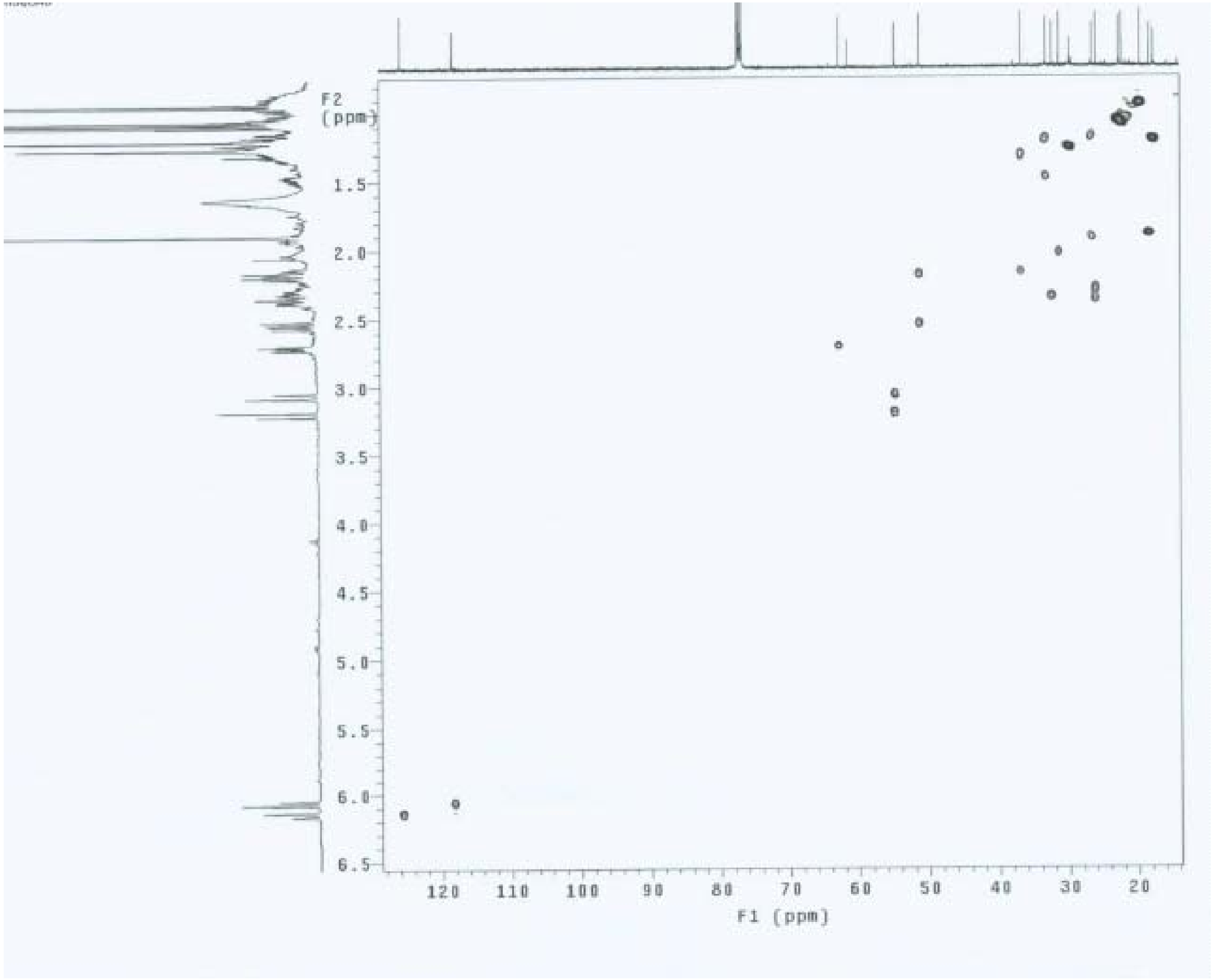

S4. HMQC spectrum of **1** in $CDCl_3$.

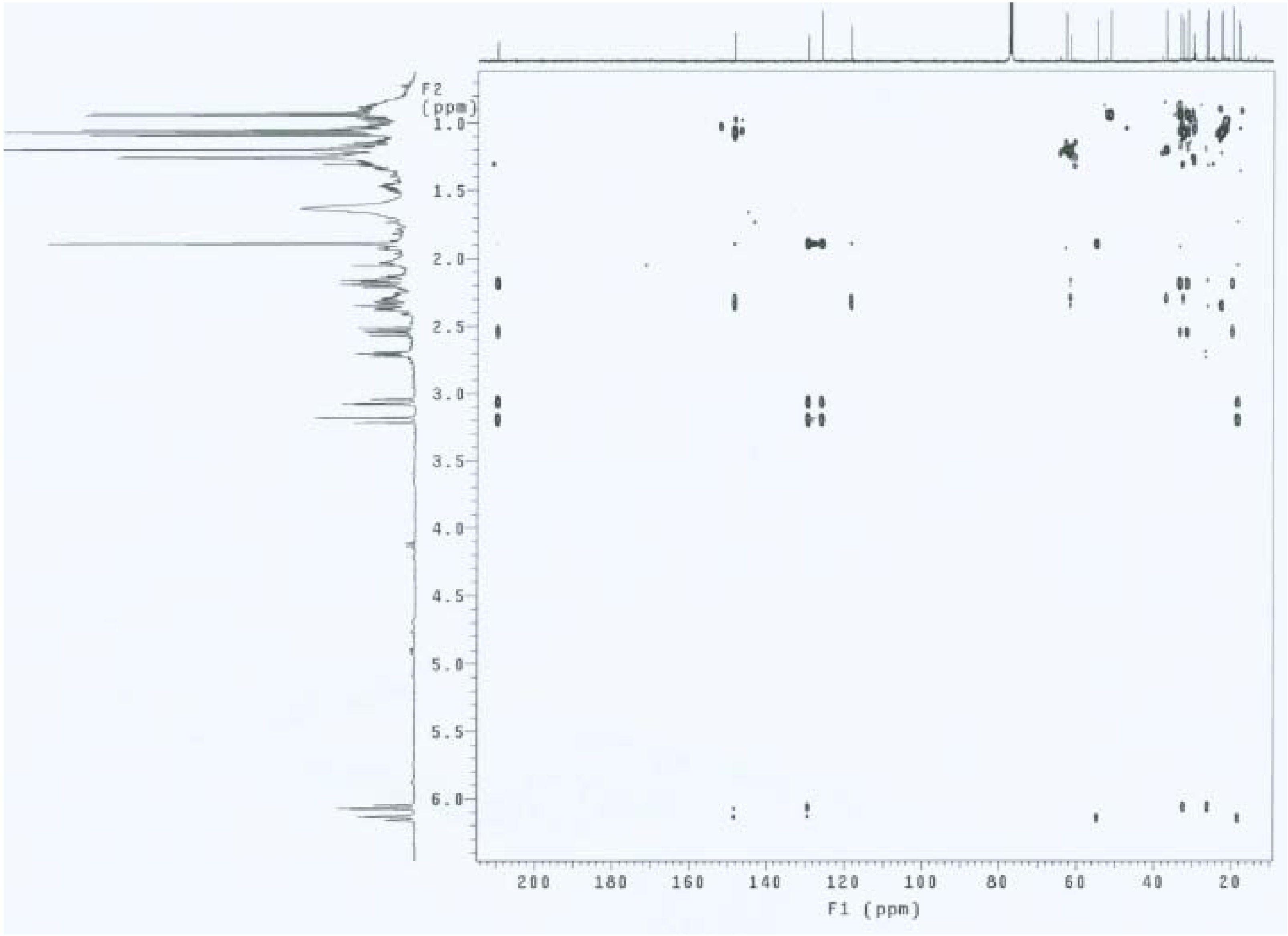

S5. HMBC-1 spectrum of **1** in $CDCl_3$

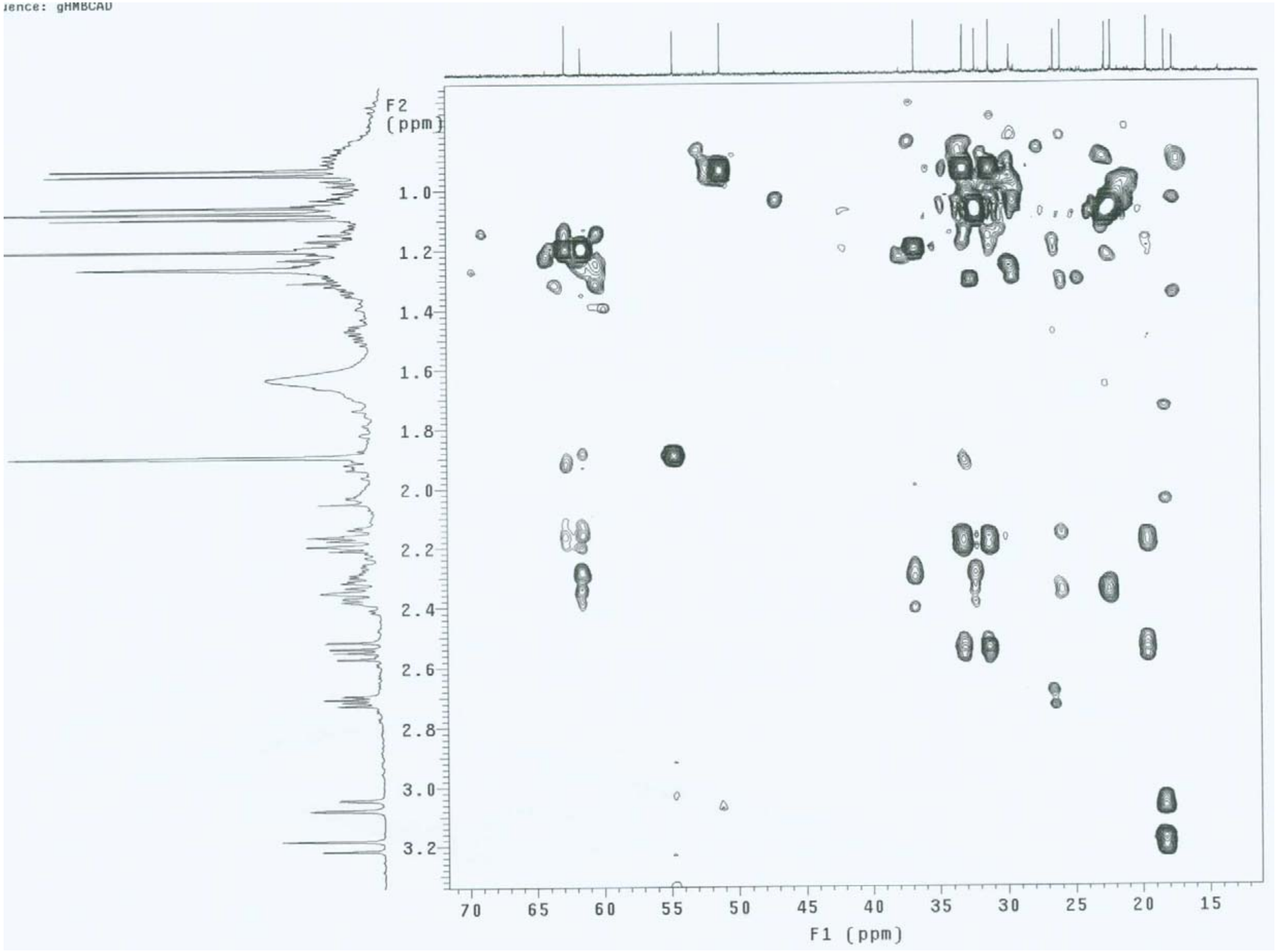

S6. HMBC-2 spectrum of **1** in $CDCl_3$

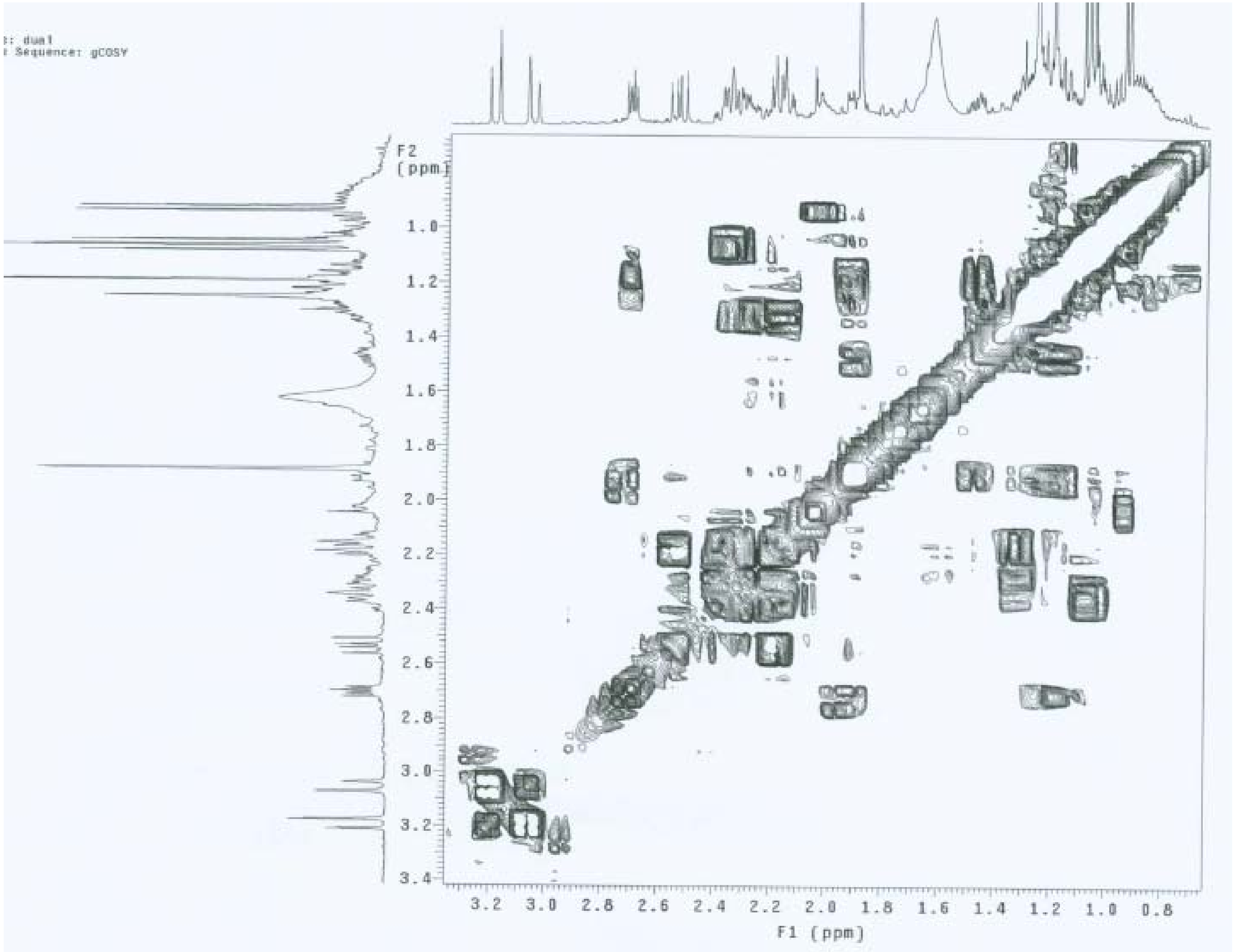

S7. H–^{1}H COSY spectrum of **1** in $CDCl_3$.
